# Foreign Languages Sound Fast: Evidence from Implicit Rate Normalization

**DOI:** 10.3389/fpsyg.2017.01063

**Published:** 2017-06-28

**Authors:** Hans Rutger Bosker, Eva Reinisch

**Affiliations:** ^1^Max Planck Institute for PsycholinguisticsNijmegen, Netherlands; ^2^Donders Institute for Brain, Cognition and Behaviour, Radboud UniversityNijmegen, Netherlands; ^3^Institute of Phonetics and Speech Processing, Ludwig Maximilian University of MunichMunich, Germany

**Keywords:** speech rate, speech segmentation, rate normalization, second language acquisition, L2 speech perception, ‘Gabbling Foreigner Illusion’

## Abstract

Anecdotal evidence suggests that unfamiliar languages sound faster than one’s native language. Empirical evidence for this impression has, so far, come from *explicit* rate judgments. The aim of the present study was to test whether such perceived rate differences between native and foreign languages (FLs) have effects on *implicit* speech processing. Our measure of implicit rate perception was “normalization for speech rate”: an ambiguous vowel between short /a/ and long /a:/ is interpreted as /a:/ following a fast but as /a/ following a slow carrier sentence. That is, listeners did not judge speech rate itself; instead, they categorized ambiguous vowels whose perception was implicitly affected by the rate of the context. We asked whether a bias towards long /a:/ might be observed when the context is not actually faster but simply spoken in a FL. A fully symmetrical experimental design was used: Dutch and German participants listened to rate matched (fast and slow) sentences in both languages spoken by the same bilingual speaker. Sentences were followed by non-words that contained vowels from an /a-a:/ duration continuum. Results from Experiments 1 and 2 showed a consistent effect of rate normalization for both listener groups. Moreover, for German listeners, across the two experiments, foreign sentences triggered more /a:/ responses than (rate matched) native sentences, suggesting that foreign sentences were indeed perceived as faster. Moreover, this FL effect was modulated by participants’ ability to understand the FL: those participants that scored higher on a FL translation task showed less of a FL effect. However, opposite effects were found for the Dutch listeners. For them, their native rather than the FL induced more /a:/ responses. Nevertheless, this reversed effect could be reduced when additional spectral properties of the context were controlled for. Experiment 3, using explicit rate judgments, replicated the effect for German but not Dutch listeners. We therefore conclude that the subjective impression that FLs sound fast may have an effect on implicit speech processing, with implications for how language learners perceive spoken segments in a FL.

## Introduction

It is a common impression that foreign languages (FLs) seem to be spoken faster than one’s own native language. This subjective impression manifests itself, for instance, in remarks of many language learners, frequently asking their interlocutors if they can please slow down. The effect has been termed the ‘Gabbling Foreigner Illusion’ by Cutler (2012, p. 338) and has attracted the attention of speech scientists for many decades (cf. Osser and Peng, 1964).

Empirical evidence for this FL effect (as it will be referred to throughout this paper) in speech rate perception has been provided with tasks in which listeners had to judge or sort the speech rate of sentences in different languages. For instance, Schwab and Grosjean (2004) presented recordings of French short stories, read at various rates, to a group of 96 native French speakers (i.e., native listeners) and a group of 96 Swiss German speakers (i.e., non-native listeners). They observed a clear FL effect in the rate judgments collected: on average, non-native listeners reported a higher speaking rate compared to the native listeners, even though both groups had been presented with the same French recordings. Moreover, the authors found a negative correlation between this FL effect and FL comprehension scores: the better the learners were able to understand the content of the stories, the smaller the FL effect (i.e., the smaller the difference in rate judgments to the native listeners).

Similar evidence has been found in a symmetrical experimental design by Pfitzinger and Tamashima (2006), who asked German and Japanese listeners to order sentences in both languages according to their perceived rate. It appeared that Japanese listeners overestimated the speech rate of German by 7.5% (relative to the German participants), and German listeners overestimated Japanese speech rate by 9.1% (relative to the Japanese participants).

Critically, the use of a symmetrical design and the presence of the FL effect in both listener groups in Pfitzinger and Tamashima (2006) suggests that its origin cannot solely be explained on the basis of differences in the rhythmic structure of the two languages. German is considered a “stress timed” language, where stressed syllables alternate with unstressed syllables (Grabe and Low, 2002). Japanese, in contrast is considered a “mora-timed” language (Ramus et al., 1999). Due to these differences in rhythm, the two languages differ in the number and nature of allowed syllable structures; for instance, German allows for more complex structures than Japanese. This in turn could have influenced how speech rate is perceived. If speech rate is measured as the number of syllables per second, rate could be expected to be higher for Japanese than German since potentially more syllables fit into a second given the simpler syllable structures in Japanese. However, despite these differences in language structure as well as potential differences in processing strategies associated with rhythm (Cutler, 2012), both listener groups judged the FL as faster.

Interestingly, empirical evidence for the FL effect has even been found in closely related language pairs, such as French and Spanish that are both considered to be “syllable timed” languages (Ramus et al., 1999). Schwab (2014) collected rate judgments from native (L1 Spanish) and non-native (L1 French) speakers of Spanish and showed that the non-native French speakers overestimated the speech rate in Spanish. Differences in rhythmic patterns between languages are hence unlikely to cause the FL effect.

This leads to the question of the psycholinguistic origin of the FL effect. One suggestion has been that it relates to speech segmentation strategies: resolving continuous speech into words is less efficient in non-native languages than in one’s native language (Cutler et al., 1983, 1986, 1989; Cutler, 2012). Language skills and knowledge are weaker in non-native listeners (Segalowitz, 2010), and as a consequence non-native listeners cannot draw on the same prosodic, phonotactic, and lexical strategies as native listeners can to efficiently extract words from continuous speech. Thus, their segmentation of continuous speech produced in a FL is slowed.

Neurophysiological support for delayed segmentation in non-native listeners has been provided by an ERP study by Snijders et al. (2007). Analyses of ERP responses to word repetitions *in isolation* revealed no difference between natives and non-natives: both groups showed a more positive ERP response to later presentations of the same word. However, when the word repetitions were embedded *in continuous speech*, ERP repetition effects were only observed in the native listeners, not in the non-native listeners. This indicates that segmentation and detection of words in continuous speech is exceptionally difficult for non-native listeners and hence indeed could relate to the FL effect.

So far, the implications of the FL effect for spoken communication have been limited to the overall impression that the listener has of the speech rate of a particular speaker. That is, researchers have only studied the FL effect by collecting *explicit rate judgments*. Participants in the studies introduced earlier were explicitly instructed to pay close attention to the speech rate in the speech materials and to provide evaluative judgments about the speech rate of a given stimulus after the stimulus had finished. Such experimental paradigms do not allow assessment of how the FL effect affects the cognitive processes involved in *online speech comprehension*. Moreover, because the judgments are provided relatively late in perceptual processing, they can be biased by many other factors such as stereotypes about how fast a certain language sounds. In fact, acoustic measures of speed of articulation have been shown to only explain 53% of the variance of explicitly perceived speed judgments (Bosker et al., 2013).

Therefore, the present study investigated whether and how the FL effect would impact online speech processing. Rather than collecting explicit rate judgments, speech rate perception was tested *implicitly* by means of the ‘rate normalization’ paradigm.

It has long been known that the perceived speech rate of a surrounding sentence can influence the perception of subsequent target words (Pickett and Decker, 1960). For instance, in the German minimal word pair *bannen* /banǝn/ “to ban” – *Bahnen* /ba:nǝn/ “tracks,” the vowel /a/ in the first syllable is short in *bannen* but longer in *Bahnen*. The perception of a vowel with a manipulated duration ambiguous between /a/ and /a:/ may be biased towards a particular interpretation depending on the perceived speech rate of the surrounding sentence (Reinisch, 2016a,b). That is, if the target vowel is presented following a fast carrier sentence, target perception is biased towards the long vowel /a:/. If it is presented in a slow carrier sentence, perception is biased towards short /a/. This effect has been taken as evidence that listeners interpret segmental durations relative to the surrounding speech rate, hence referred to as ‘rate normalization.’ The measure can be taken as measuring ‘implicit’ rate perception since listeners are asked to identify a target word rather than directly judge the rate of the context.

The present study adapted the ‘rate normalization’ paradigm to investigate implicit speech rate perception in a FL. Specifically, we asked whether a ‘rate normalization’ context effect (i.e., fast speech biasing perception towards a long vowel /a:/) may be observed when the context is not actually faster but simply spoken in a FL.

Note that a previous study (Bosker et al., 2017) has used implicit rate normalization to demonstrate effects of cognitive load on the perception of speech rate. In that study, carrier sentences were shown to be perceived as faster when listeners were taxed by a simultaneously presented difficult visual search task. The same principle may apply to the perception of a FL: words in a FL are harder to segment out of the continuous speech stream (Snijders et al., 2007), thus taxing the perceptual system, and consequently inducing a higher perceived speech rate.

To test the FL effect, we adopted a fully symmetrical design, with parallel experiments involving two listener groups listening to two different languages. The languages studied here were German and Dutch because both languages have a phonological /a-a:/ vowel duration contrast (for details, see Method), allowing for comparison of /a-a:/ categorization across the two languages. Note that, despite related vocabulary, German and Dutch are not mutually comprehensible without explicit focus or prior training. Importantly, the use of two closely related languages with similar grammar, syllable structures, and rhythm, allowed for maximal control of these structural factors while only varying the language.

If the FL effect (i.e., the impression that FLs sound fast) does not only impact explicit evaluative judgments but also the online processing of speech, we may find that German listeners report more long target vowels (i.e., /a:/) after Dutch carrier sentences (a language unknown to them) than after rate matched German sentences (their native language). The opposite should hold for Dutch listeners (i.e., German as their FL should sound faster). By using two highly-related languages the presence of a FL effect would suggest that it is indeed the knowledge of the language that drives the effect.

Moreover, along these lines and based on the studies by Schwab and Grosjean (2004) and Schwab (2014), we would expect this Language effect to interact with listeners’ ability to understand the FL: listeners who understand more words in the FL – here also referred to as higher proficiency in the FL^1^ – should show less of a FL effect.

## Experiment 1

### Method

#### Participants

A group of native Dutch participants (*N* = 27; 18 females, 9 males; *M*_age_ = 23) with little knowledge of German was recruited from the Max Planck Institute’s participant pool. Another group of native German participants (*N* = 23; 15 females, 8 males; *M*_age_ = 23) with little knowledge of Dutch was recruited. Of these 23 German participants, 20 participants were recruited from the student population at the University of Munich; the remaining three participants were recruited from the Max Planck Institute’s participant pool. All participants reported to have normal hearing and gave written informed consent as approved by the Ethics Committee of the Social Sciences department of Radboud University (project code: ECSW2014-1003-196). Overall proficiency in the FL was assessed by means of self-reported listening skills. Participants rated “how well you understand spoken [Dutch/German]” on a scale from 1 (“absolutely no understanding”) to 7 (“very much understanding”): *M*_Dutch Group_ *(SD)* = 2.9 (1.0); *M*_German Group_ = 0.8 (1.4); *t*(48) = 6.158, *p* < 0.001.

#### Design and Materials

A female German-Dutch bilingual speaker (bilingual from birth; no accent in either language) was recorded producing 30 sentences in German and 30 sentences in Dutch. The Dutch sentences were paraphrases of the German sentences, matching in number of syllables (see Appendix). None of the sentences contained any /a/ or /a:/ vowels since these made up the critical contrast for the targets. Each sentence was recorded with one of three minimal pairs in sentence-final position, selected to be non-words in either language: *faft – faaft, fapt – faapt, fap – faap*.

From these recordings, carrier sentences (i.e., all speech up to target onset) were excised. Using PSOLA in Praat (Boersma and Weenink, 2016), the total duration of each Dutch–German sentence pair was set to the mean duration of that pair. That is, the speaking rate of each sentence pair was equalized. Since the bilingual speaker produced the sentences at a rather slow speech rate, these (duration matched) carrier sentence pairs formed the slow condition in the experiments. Linear compression by a factor of 0.6 resulted in the fast condition.

Target non-words were manipulated with the aim to create an /a-a:/ duration continuum that is categorized similarly by Dutch and German listeners. In German, the contrast between /a/ (e.g., *bannen* “to ban”) and /a:/ (e.g., *Bahnen* “tracks”) is cued by temporal properties alone (i.e., without consistent co-variation of spectral properties; Jessen, 1993; Pätzold and Simpson, 1997; Reinisch, 2016a,b), with /a/ having a shorter duration than /a:/. In Dutch, the vowel contrast is cued by both spectral (/ɑ/ has relatively low formant values, particularly F2) and temporal properties (/ɑ/ has a relatively short duration; Adank et al., 2004; Escudero et al., 2009; Reinisch and Sjerps, 2013; Bosker, 2017a; Bosker et al., 2017). Because temporal variation influences both German and Dutch listeners in /a-a:/ categorization, a duration continuum from /a/ to /a:/ was created, while spectral properties of all steps on the continuum were controlled to be ambiguous for all listeners.

One particular /a:/ vowel token was selected for manipulation using Burg’s LPC method and PSOLA in Praat. A two-dimensional spectral-temporal continuum was created around the average F2 and duration values of the speaker in both languages. Based on a pretest of this two-dimensional continuum with Dutch (*N* = 15) and German (*N* = 12) listeners (none participated in any of the other experiments), the most ambiguous spectral values (F1 = 655 Hz; F2 = 1280 Hz) were selected to be used in a five-step duration continuum from 120 to 160 ms in steps of 10 ms for the main experiments. These five spectrally ambiguous vowel tokens were categorized similarly by Dutch (average % /a:/ categorization: 55%) and German listeners (average % /a:/ categorization: 51%). This observation was confirmed with a Generalized Linear Mixed Model (GLMM) with a logistic linking function that was fit with the predictors Vowel Duration, Listener Group, their interaction and with Participant as a random factor (β = 0.299; *p* > 0.35). These vowel tokens were spliced into three consonantal frames (/f_p/; /f_pt/; /f_f/) resulting in 15 target non-words.

#### Procedure

In Experiment 1, each trial started with the presentation of a fixation cross. After 500 ms, the carrier sentence was presented, followed by a silent interval of 100 ms, followed by the target. At target offset, the fixation cross was replaced by a screen with two response options, one on the left, one on the right (position of /a/-/a:/ non-words counter-balanced across participants). Participants entered their response as to which of the two response options they heard (*fap* or *faap*, etc.) by pressing “1” for the option on the left, or “0” for the option on the right. After their response (or timeout after 4 s), the screen was replaced by an empty screen for 500 ms, after which the next trial was initiated.

Language (native vs. foreign) was blocked, with order counter-balanced across participants. Participants were presented with 15 carriers in their L1 and the other 15 carriers in their FL to avoid carrier familiarity effects across blocks. One language block included 150 randomized trials: 15 carriers × 2 rates × 5 vowel steps; the particular consonantal frame was selected using a Latin Square design. Participants were allowed to take a break in between language blocks.

In order to assess participants’ recognition accuracy of the FL materials, participants were asked to translate the first 15 trials of the FL block into their L1. These first 15 trials all involved unique carrier sentences that participants had not heard before. Participants entered their translation after having given their categorization response; that is, they typed out their translation on the computer keyboard. Participants’ recognition accuracy was assessed by percentage of keywords correct. In order to match the L1 and FL blocks, participants also transcribed the first 15 trials of the L1 block.

### Results

The Dutch group performed significantly better at translating German than the German group did in translating Dutch (in % keywords correct): *M*_Dutch Group_ *(SD)* = 54.3 (36.1); *M*_German Group_ *(SD)* = 30.9 (33.2); *t*(724) = 8.892, *p* < 0.001.

**FIGURE 1:**
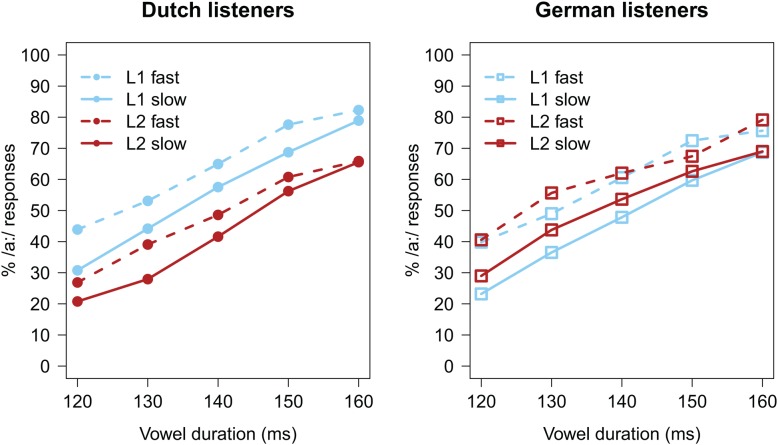
Average categorization data (in % /a:/ responses) of Experiment 1. **(Left)** Data from the Dutch listener group; **(Right)** data from the German listener group.

Before analyzing the categorization data, trials with missing categorization responses (*n* = 53; <1%) were excluded from analyses. Categorization data, calculated as the percentage of /a:/ responses (% /a:/), are presented in **Figure 1**, separately for each listener group. As expected, an increase in target vowel duration led all listeners to report more /a:/ responses (all lines have a positive slope). The difference between the solid and dashed lines indicates an influence of the carrier’s speech rate, with faster speech rates (dashed lines) biasing perception towards the long vowel /a:/. Importantly, differences between the blue and red lines indicate effects of the precursor’s language, and it would seem that the language effect is in opposite directions for the two listener groups.

We quantified these effects using a GLMM (Quené and Van den Bergh, 2008) with a logistic linking function as implemented in the lme4 library, version 1.0.5 (Bates et al., 2015) in R (R Development Core Team, 2012). The dependent variable was response /a:/ (coded as 1) or /a/ (coded 0). Fixed effects were Vowel Duration (continuous predictor, centered, and scaled around the mean), Carrier Rate (categorical predictor, with slow speech rate coded as -0.5 and fast speech rate as +0.5), Language (categorical predictor, with L1 coded as -0.5 and FL coded as +0.5), Listener Group (categorical predictor, with Dutch coded as -0.5 and German coded as +0.5), and the interaction between Language and Listener Group. The use of deviation coding of two-level categorical factors (i.e., coded with +0.5 and -0.5) allows us to test main effects of these predictors, since with this coding the grand mean is mapped onto the intercept. Participant and Carrier Item were entered as random factors with by-participant and by-carrier random slopes for Carrier Rate and Language (Barr et al., 2013). A more extended model also including random slopes for Listener Group failed to converge.

The GLMM revealed a significant effect of Vowel Duration (β = 0.792, *z* = 38.430, *p* < 0.001), with longer vowel durations increasing the percentage of /a:/ responses. The effect of Carrier Rate (β = 0.483, *z* = 5.700, *p* < 0.001) indicated that the faster the carrier’s speech rate, the higher the percentage of /a:/ responses. An effect of Language (β = -0.343, *z* = -2.860, *p* = 0.004) indicated that there was a *lower* percentage of /a:/ responses when the vowel was preceded by a FL carrier. However, an interaction between Language and Listener Group (β = 0.976, *z* = 4.280, *p* < 0.001) revealed that this only held for the Dutch group; the German group showed an opposite pattern, with a *higher* percentage of /a:/ responses after FL carriers. Taking categorization differences as indices of perceived rate, this suggests that, while for Dutch listeners foreign speech appeared to sound slower than their native language, Germans did show the expected pattern that FL speech sounds fast.

**FIGURE 2:**
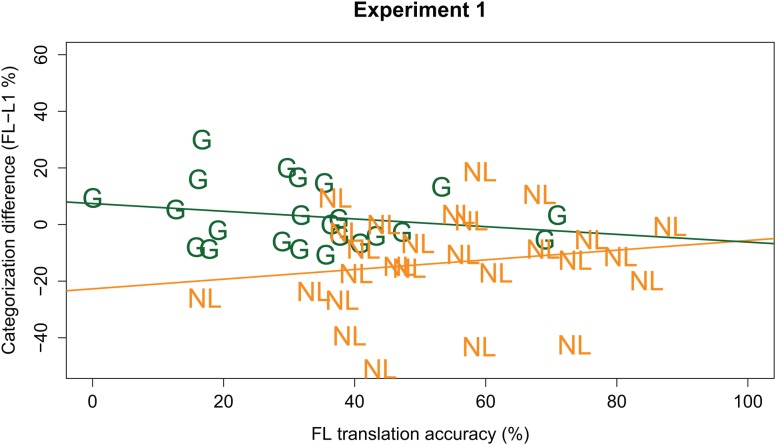
Individual participants’ foreign language (FL) effect (y-axis; calculated as % /a:/ responses in FL *minus* L1; positive values indicate a higher percentage /a:/ responses in the FL) plotted against individual participants’ translation accuracy (x-axis; in % keywords correct) in the FL. German participants are indicated by green “G”; Dutch participants by orange “NL.” The green line gives the regression line for the German group; the orange line gives the regression line for the Dutch group.

In order to test whether the Language effects observed were modulated by participants’ ability to understand the FL, the GLMM was extended with the predictor Translation Accuracy (continuous predictor, centered, and scaled around the mean), and the interactions between Translation Accuracy and other fixed effects. This extended GLMM modeled the data marginally better [χ^2^(4) = 8.339, *p* = 0.079] than the initial model reported above. It revealed similar effects as the previous model (i.e., effects of Vowel Duration, Carrier Rate, Language, and Language × Listener Group interaction); however, it also showed a three-way interaction between Language, Listener Group, and Translation Accuracy (β = -0.245, *z* = -2.680, *p* = 0.007). *Post hoc* analyses, run on the data from the Dutch and German listener groups separately, revealed that this three-way interaction is explained by a negative effect of Translation Accuracy on the Language effect in the German group (β = -0.130, *z* = -2.029, *p* = 0.042), but a positive effect of Translation Accuracy on the Language effect in the Dutch group (β = 0.128, *z* = 1.989, *p* = 0.047; see **Figure 2**). This suggests that, for the German group, the better the Germans understood the FL, the less of a difference there was between their native and FL categorization patterns. That is, the more ‘proficient’ the German listener, the less fast Dutch sounds to them (in line with our predictions). However, the *post hoc* analyses for the Dutch group suggest that the better a Dutch listener understands German, the faster German sounds (contrary to our predictions).

### Discussion

Experiment 1 found partial support for the hypothesis that a FL sounds fast, with consequences for online speech processing. German listeners indeed reported a higher percentage of long vowel (/a:/) responses when the target vowel followed a FL carrier sentence compared to a (rate matched) L1 carrier sentence. This suggests that when the German participants listened to Dutch (to them, a FL), they perceived the carrier sentence as relatively fast, biasing their perception of subsequent ambiguous vowels towards the long vowel /a:/; similar to how actually (acoustically) fast speech biases perception towards /a:/. Moreover, a three-way interaction indicated that this Language effect in the German group was modulated by their ability to comprehend the Dutch sentences: the better they understood the sentences, the less fast they sounded (i.e., the fewer /a:/ responses).

However, the Dutch participant group showed the opposite pattern. Where the German group reported more /a:/ responses after listening to a FL (Dutch) carrier sentence, the Dutch participants reported *fewer* /a:/ responses after listening to their FL (German). This would suggest that, to Dutch listeners, German actually sounds slow relative to Dutch, in contrast to our predictions. Moreover, an unexpected three-way interaction suggested that the better the Dutch listeners understood German sentences, the faster it sounded to them.

In Experiment 1, the German and Dutch carrier sentences were matched in their temporal characteristics: both members of each sentence pair had the same number of syllables and the exact same sentence duration. However, the spectral properties of the carrier sentences were not controlled. Note that, although Dutch and German are closely related languages and we used close paraphrases of the sentences in both languages (see Appendix), the vowels occurring in the Dutch and German sentences differed (i.e., as part of the different vocabularies). This difference in vowels meant that the average formant values of the Dutch and German carrier sentences differed despite the fact that the same bilingual speaker had produced the two sentence sets. Specifically, the Dutch average F2 was lower (F2 = 1739 Hz [*SD* = 149]) than the German average F2 (F2 = 1865 Hz [*SD* = 143]; *t*(29) = -4.082; *p* < 0.001).

Considering the fact that the Dutch /ɑ-a:/ contrast is also cued by spectral properties, its perception is sensitive to the spectral properties in the sentence context as well. For instance, Dutch listeners may be biased to reporting *fewer* /a:/ targets by raising the average F2 in the surrounding sentence (Reinisch and Sjerps, 2013; Bosker et al., 2017). This process, known as spectral normalization (Sjerps et al., 2011), may potentially explain why, in Experiment 1, the Dutch listeners reported *fewer* /a:/ responses after the German carrier sentences with a relatively higher average F2. The different vowels in the German sentences, with a relatively high average F2, may have induced spectral normalization in the Dutch listeners, biasing their perception of the target vowels towards /ɑ/. In contrast, in German, the /a-a:/ contrast is a temporal one that is likely not sensitive to spectral context effects. Therefore, it could be the case that the difference in formants between the Dutch and German carrier sentences influenced the Dutch group (not the German group). Experiment 2 was designed to investigate this potential explanation by matching the average second formant values of the Dutch and German sentences.

## Experiment 2

### Method

#### Participants

Two new groups of native Dutch (*N* = 24; 20 females, 4 males; *M*_age_ = 21; recruited at the Max Planck Institute) and native German participants (*N* = 24; 15 females, 9 males; *M*_age_ = 26; recruited at the University of Munich) were recruited according to the same criteria as previously and participated with written informed consent. Overall proficiency in the FL was assessed by means of self-reported listening skills on a scale from 1 to 7: *M*_Dutch Group_ *(SD)* = 2.3 (0.8); *M*_German Group_ = 0.5 (0.8); *t*(44) = 7.912, *p* < 0.001.

#### Design and Materials

The design of Experiment 2 was identical to that of Experiment 1, except that the average spectral characteristics of the carrier sentences were also matched across languages (after duration matching, to create the slow condition, and before linear compression, to create the fast condition). For each carrier, source and filter models of all vowels were created using Burg’s LPC method in Praat. Second formant values were shifted by -20, -10, 0, +10, +20% in each vowel. After source and filter recombination, F1 and F2 frequencies of the resulting manipulated carrier sentences were inspected. For each sentence pair, the best matching spectral manipulation was selected. For instance, the original carrier sentence 13 (see Appendix) had an average F2 of 1686 Hz in Dutch and 1931 Hz in German. The best matching spectral pairing involved the +10% version in Dutch (F2 = 1817 Hz) and the -10% version in German (F2 = 1872 Hz). The resulting spectrally matched Dutch and German materials (average Dutch F2 = 1783 Hz [*SD* = 135]; average German F2 = 1784 Hz [*SD* = 143]; *t*(29) = -0.219; *p* > 0.8) were afterward compressed by 0.6 to create the fast condition for Experiment 2.

### Results

Similar to Experiment 1, the Dutch group performed significantly better at translating (their FL) German than the German group did in translating Dutch (in % keywords correct): *M*_Dutch Group_ *(SD)* = 43.1 (34.7); *M*_German Group_ *(SD)* = 28.7 (31.2); *t*(709) = 5.834, *p* < 0.001.

**FIGURE 3:**
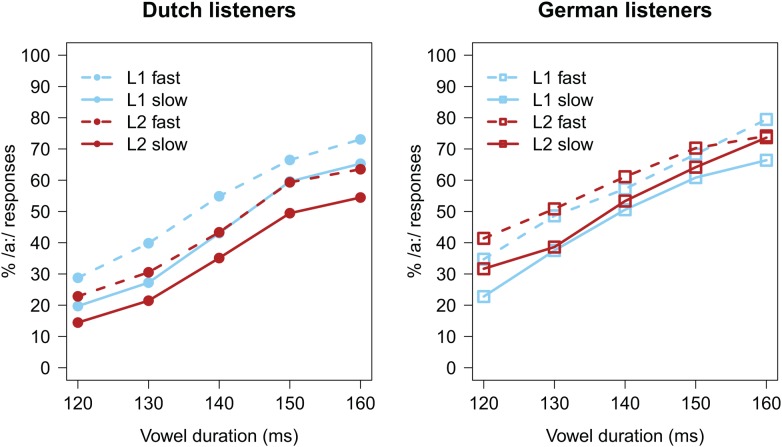
Average categorization data (in % /a:/ responses) of Experiment 2. **(Left)** Data from the Dutch listener group; **(Right)** data from the German listener group.

Trials with missing categorization responses (*n* = 17; <1%) were excluded from analyses. Categorization data, calculated as the percentage of long vowel responses (% /a:/), are presented in **Figure 3**, separately for each listener group. Similar to Experiment 1, increasing the target vowel duration led all listeners to report more /a:/ responses (all lines with positive slopes). The difference between the solid and dashed lines indicates an influence of the carrier’s speech rate, with faster speech rates (dashed lines) biasing perception towards the long vowel /a:/. Importantly, differences between the blue and red lines indicate effects of the precursor’s language, and, like Experiment 1, it would seem that the language effect is in opposite direction in the two panels.

These effects were quantified using a GLMM with a logistic linking function, and identical structure as the one used for analyzing the data from Experiment 1. This model revealed a significant effect of Vowel Duration (β = 0.853, *z* = 39.550, *p* < 0.001), with longer vowel durations increasing the percentage of /a:/ responses. The effect of Carrier Rate (β = 0.510, *z* = 7.010, *p* < 0.001) indicated that the faster the carrier’s speech rate, the higher the percentage of /a:/ responses. No overall effect of Language was observed (β = -0.177, *z* = -1.220, *p* > 0.2). However, an interaction between Language and Listener Group (β = 0.807, *z* = 2.860, *p* = 0.004) revealed that, in the German group, there was a *higher* percentage of /a:/ responses after FL carriers (compared to L1 carriers). This suggests, similar to Experiment 1, that, while for Dutch listeners FL speech appeared to sound slower than their L1, Germans did show the pattern that FL speech sounds fast.

Note that when comparing the left panels of **Figures 1**, **3**, it would appear as if the Language effect in the Dutch group was smaller in Experiment 2 than in Experiment 1. In order to test whether the spectral manipulation in Experiment 2 had changed the results relative to Experiment 1, datasets from the Dutch participants of both experiments were combined. This combined dataset was tested using a GLMM with identical structure as the previous one, except that it was extended with the categorical predictor Experiment (with Experiment 1 coded as -0.5 and Experiment 2 coded as +0.5) and the interactions between Experiment and the other fixed effects. This GLMM revealed a significant interaction between Language and Experiment (β = 0.231, *z* = 2.920, *p* = 0.003), indicating that the Language effect in the Dutch group in Experiment 2 was significantly smaller than the Language effect in the Dutch group in Experiment 1.

**FIGURE 4:**
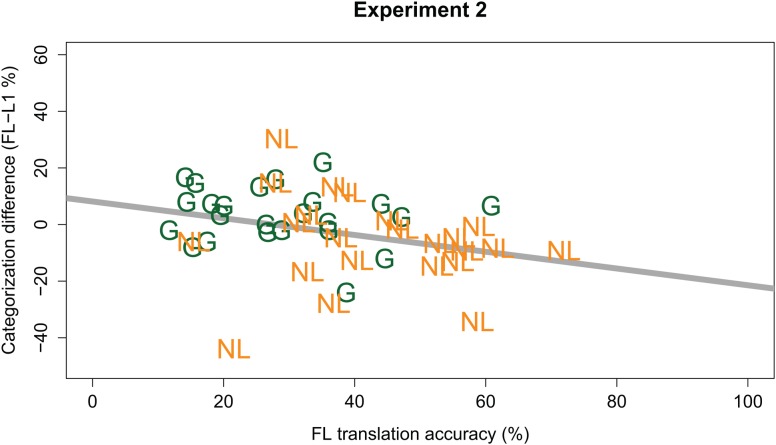
Individual participants’ FL effect (y-axis; calculated as % /a:/ responses in FL *minus* L1; positive values indicate a higher percentage /a:/ responses in the FL) plotted against individual participants’ translation accuracy (x-axis; in % keywords correct) in the FL. German participants are indicated by green “G”; Dutch participants by orange “NL.” The bold gray line gives the regression line for the combined data from both groups.

Similar to Experiment 1, we also investigated whether any Language effects were modulated by participants’ ability to understand the FL sentences. Therefore, the initial GLMM of Experiment 2 was extended with the predictor Translation Accuracy (continuous predictor, centered, and scaled around the mean), and the interactions between Translation Accuracy and other fixed effects. This extended GLMM modeled the data marginally better than the one without Translation Accuracy [χ^2^(4) = 9.217, *p* = 0.056]. It revealed an additional two-way interaction between Language and Translation Accuracy (β = -0.111, *z* = -2.340, *p* = 0.019). No three-way interaction between Language, Listener Group, and Translation Accuracy was observed. As shown in **Figure 4**, the two-way interaction indicated that, across both Listener Groups, any Language effect was modulated by Translation Accuracy. The negative sign of the interaction helps in interpreting this modulating effect; that is, the better participants understood the FL sentences (i.e., higher Translation Accuracy), the slower the FL sounded (i.e., as evidenced by *fewer* /a:/ responses).

### Discussion

Results from Experiment 2 again showed partial support for the hypothesis that a FL sounds fast, and again primarily in the German group. German listeners reported more /a:/ responses after FL carriers than after rate and spectrally matched L1 carriers, similar to Experiment 1. On average, the Dutch group showed the opposite pattern, similar to the results from Experiment 1: FL carriers resulted in *fewer* /a:/ responses, hence FL speech supposedly sounded slower than L1 speech. However, a comparison with Experiment 1 revealed that this effect in the Dutch group (i.e., in opposite direction to our hypothesis) was considerably weaker in Experiment 2 relative to Experiment 1. This reduction may be attributed to the spectral matching procedure in Experiment 2.

Moreover, interactions with FL proficiency metrics showed that better ability to understand the FL sentences reduced the Language effect. This suggests that FLs sound fast particularly for low-proficient listeners and that this effect is weaker the better listeners are able to understand the FL. Note that this modulating effect of FL proficiency held for both listener groups, regardless of the absolute difference between L1 and FL categorization.

Although part of the effect that Dutch listeners showed an unexpected pattern for Language could be explained by spectral effects, the question remains why the Dutch speech materials consistently induced a higher percentage of long vowel responses across groups and experiments. In Experiment 2, sentences in the two languages were matched on speaker, the number of syllables, overall sentence duration and certain spectral characteristics that could have influenced categorization responses. One possible remaining explanation may involve interactions between the FL effect and more general expectations about the habitual speech rates of talkers of a particular language.

Cross-linguistic studies of speech rate show that German is typically produced with a relatively higher average syllable rate of approximately six syllables a second (Pellegrino et al., 2011) compared to Dutch with an average syllable rate of approximately four syllables a second (Quené, 2008 though note that these two studies used different speech elicitation tasks). If, based on prior exposure, our Dutch participants happened to have a stereotypical expectation that German talkers typically speak rather fast, this expectation may have contrasted with the actually observed speech rates in our experimental materials (matched in rate to Dutch speech). As a consequence, the German speech in our experiments may have sounded relatively slow to the Dutch listeners (as compared against their stereotypical expectations), potentially explaining why the Dutch listeners reported *fewer* /a:/ responses after German carrier sentences. Note that such an account would be in line with findings by Bosker and Reinisch (2015) who found that, although non-native (i.e., foreign-accented) speech is typically slower than native speech, rate matched non-native speech is actually perceived as faster.

Any potential stereotypical expectations about the speech rate of a particular language would be expected to show up when participants are asked to explicitly rate the speech rate of different languages. Therefore, Experiment 3 was designed to test whether the rate of the German and Dutch carrier sentences was perceived differently in an experimental task involving *explicit rate judgments*.

## Experiment 3

### Method

#### Participants

Two new groups of native Dutch participants (*N* = 20; 14 females, 6 males; *M*_age_ = 35; recruited at Max Planck Institute) and native German participants (*N* = 22; 14 females, 8 males; *M*_age_ = 26; recruited at University of Munich) were recruited according to the criteria of the previous experiments and participated with written informed consent. FL proficiency was assessed by means of self-reported listening skills on a scale from 1 to 7: *M*_Dutch Group_ *(SD)* = 3.2 (1.1); *M*_German Group_ = 1.7 (0.8); *t*(39) = 4.728, *p* < 0.001.

#### Design and Materials

Experiment 3 used the materials from Experiment 2. However, in Experiment 3 only carrier sentences were used, not the target materials. Recall that the ‘slow’ condition in the previous experiments was the result of setting the duration of each sentence to the mean of each sentence pair (see Methods of Experiment 1). The ‘fast’ condition was created by linearly compressing the ‘slow’ condition by a factor of 0.6. For Experiment 3, five additional rate conditions (next to the ‘fast’ and ‘slow’ conditions) were created by linear compression/expansion of the ‘slow’ condition using PSOLA in Praat. Three of these were chosen to fall in between the slow and fast conditions from Experiments 1 and 2 (factors of 0.85; 0.75; 0.66) and two to fall outside their scope (factors of 1.2 and 0.55).

#### Procedure

Participants in Experiment 3 were presented the carrier sentences (i.e., without target intervals) at seven different rates, with instructions to rate the speech rate of the sentence on a scale from 1 (“very slow”) to 9 (“very fast”). Participants heard half (*n* = 15) of the carriers in their L1 and the other half in their FL (language blocked; order counter-balanced across participants; i.e., the overall design matched Experiments 1 and 2). Within each language block, each carrier-rate combination was presented twice, in random order. In Experiment 3, no translations were asked from participants; only speed ratings were collected.

### Results

**FIGURE 5:**
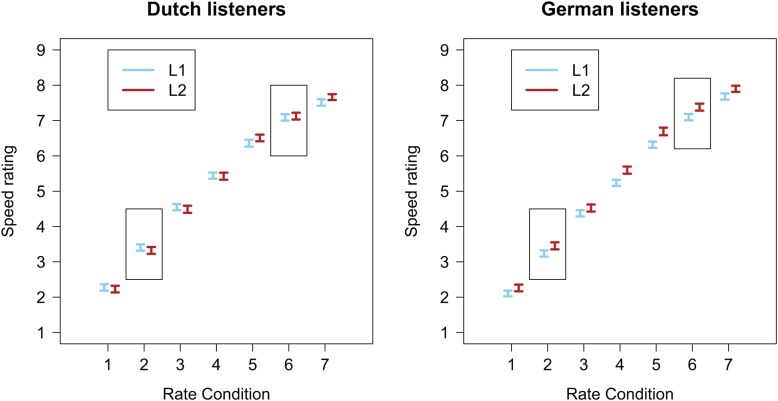
Average speed ratings of Experiment 3 (error bars show standard errors). **(Left)** Data from the Dutch listener group; **(Right)** data from the German listener group. The rectangles indicate the two rate conditions used in Experiments 1 and 2.

Rating data, with 1 meaning “very slow” and 9 meaning “very fast,” are presented in **Figure 5** separately for each listener group. The difference between the blue and red lines indicates an effect of Language, which only seems to be present in the German group: Dutch would seem to sound faster than (rate matched) German sentences.

Effects were quantified using a Linear Mixed Model (LMM). The dependent variable was rating on a scale from 1 to 9. Fixed effects were Rate (continuous predictor, scaled and centered around the mean), Language (categorical predictor, with L1 coded as -0.5 and FL coded as +0.5), Listener Group (categorical predictor, with Dutch coded as -0.5 and German coded as +0.5), and the interaction between Language and Listener Group. Participant and Carrier Item were entered as random factors with by-participant and by-carrier random slopes for Rate and Language (Barr et al., 2013). Statistical significance was asessed at the 0.05 significance level by checking whether *|t|* > 2 (Baayen, 2008).

This model revealed a significant effect of Rate (β = 1.862, *SE* = 0.294, *t* = 6.330) indicating that the faster the speech rate, the higher the rating. An effect of Language (β = 0.139, *SE* = 0.047, *t* = 2.970) revealed that, based on the grand mean calculated across the two Listener Groups, FL speech received higher speed ratings than (rate matched) L1 speech. However, an interaction between Language and Listener Group (β = 0.250, *SE* = 0.094, *t* = 2.670) showed that this Language effect was really only present in the German group.

**FIGURE 6:**
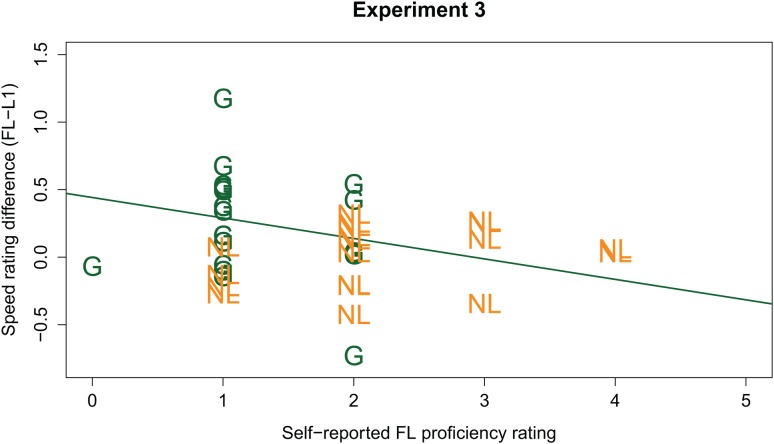
Individual participants’ FL effect (y-axis; calculated as FL speed ratings *minus* L1 ratings; positive values indicate a higher speed rating in the FL) plotted against individual participants’ self-reported FL knowledge (x-axis). German participants are indicated by green “G”; Dutch participants by orange “NL.” The green line gives the regression line for the German group.

Similar to the previous experiments, FL proficiency metrics were added to the LMM to test whether the ability to understand the FL sentences modulates the Language effect. Because translations had not been collected in Experiment 3, we added the self-reported FL ratings (on a scale from 1 to 7; continuous predictor, scaled and centered around the mean), and interactions between the self-reported FL ratings and other fixed effects to the LMM^2^. This extended LMM modeled the data significantly better than the simpler model [χ^2^(4) = 1201.4, *p* < 0.001]. It revealed an additional three-way interaction between Language, Listener Group, and the self-reported FL ratings (β = -0.237, *SE* = 0.053, *t* = -4.500). *Post hoc* analyses, run on the data from the Dutch and German listener groups separately, revealed that this three-way interaction is explained by a negative effect of self-reported FL ratings on the Language effect in the German group (β = -0.240, *SE* = 0.047, *t* = -5.060; see **Figure 6**). This suggests that, for the German group, the higher the Germans judged their own FL skills, the less of a difference there was between their native and FL speed ratings. That is, the more ‘proficient’ the German listener, the less fast Dutch sounds to them (in line with our predictions). No modulating effect of self-reported FL ratings was found in the *post hoc* analyses for the Dutch group (*t* < 1).

### Discussion

The results from Experiment 3 again provide partial support for our hypothesis that a FL sounds fast. Collection of explicit rate judgments revealed that German listeners indeed rated Dutch (a FL) as faster than (rate matched and spectrally matched) L1 speech. Moreover, a three-way interaction indicated that this Language effect in the German group was modulated by their self-reported FL ‘proficiency’: the higher their self-rated FL proficiency, the less fast FL speech sounds.

However, the Language effect was again only observed in the German group, not in the Dutch group, where we could not find evidence to support our hypothesis that FL speech sounds fast. Nevertheless, the null effect in the Dutch group (1) supports our efforts to match the rates of the two sets of carrier sentences; (2) does not support the proposal that any stereotypical expectations about the habitual speech rate of talkers of German interacted with the Language effect in previous experiments.

## General Discussion

The present study investigated the FL effect, also known as ‘Gabbling Foreigner Illusion’ (Cutler, 2012): the common impression of many listeners that FLs tend to sound faster than one’s native language. Previous studies using *explicit rate perception* paradigms (e.g., rate judgments) have shown empirical support for this FL effect (Schwab and Grosjean, 2004; Pfitzinger and Tamashima, 2006; Schwab, 2014). However, these studies only show that the FL effect impacts listeners’ evaluative impressions of the speech rate of a foreign speaker.

The present study investigated whether the FL effect would actually impact the cognitive processes involved in *online speech perception*. To do so, Experiments 1 and 2 studied *implicit rate perception* using the ‘rate normalization’ paradigm. Context sentences with a fast speech rate have been shown to bias the perception of a subsequent temporal vowel contrast (i.e., short /a/ vs. long /a:/) towards the long vowel (Bosker, 2017b; Bosker and Kösem, 2017). That is, listeners report on vowel identity that is implicitly influenced by the rate of the context rather than making explicit rate judgments. We asked whether listening to a FL that is not actually (acoustically) fast could bias perception of subsequent ambiguous /a/-/a:/ vowels towards the long vowel /a:/ as well (relative to a native language context). This would suggest that the FL is *perceived* to be fast. This question was addressed using a fully crossed experimental design (i.e., Dutch and German participants listening to both German and Dutch speech).

Experiment 1, using temporally matched Dutch and German carrier sentences, indeed revealed that German listeners reported more long-vowel (/a:/) responses after Dutch carriers than after (rate matched) German carriers, suggesting that Dutch, to them a FL, actually sounded fast. However, Dutch listeners showed an opposite Language effect: Dutch listeners reported *fewer* long vowel responses after carriers in the FL German than after Dutch carriers, suggesting that to our Dutch participants, German actually sounded *slow*.

Experiment 2 revealed that this unexpected (i.e., opposite to our predictions) Language effect in the Dutch group could partially be explained by normalization for the spectral characteristics of the Dutch and German sentences in the Dutch group. In Experiment 2, both the temporal and spectral characteristics of the German and Dutch sentences were matched. Data from a new sample of German participants replicated the findings from Experiment 1: German listeners reported more long-vowel (/a:/) responses after Dutch carriers than after temporally and spectrally matched German carriers, suggesting that Dutch (their FL) actually sounded fast to them. Spectral characteristics of the sentences did not influence this effect. At the same time, data from a new sample of Dutch participants showed that the unexpected Language effect was significantly reduced in Experiment 2 relative to Experiment 1. Nevertheless, FL German carrier sentences still elicited *fewer* long-vowel responses in the Dutch group relative to the native Dutch carrier sentences.

Experiment 3 showed that this unexpected Language effect in the Dutch group could not be explained by stereotypical expectations in Dutch listeners about average speech rates in German. In Experiment 3, we collected explicit speech rate judgments of the German and Dutch sentences and observed no difference in how Dutch participants evaluated Dutch and temporally and spectrally matched German speech. However, we also observed – in line with our predictions and replicating the results from Experiments 1 and 2 – that German listeners perceive the FL Dutch as faster than their native language German. The Dutch sentences received higher speed ratings from the German listeners than the German sentences.

Taken together, the present experiments demonstrated support for the FL effect throughout our three German participant samples. German listeners perceive Dutch carrier sentences (to them, a FL) as faster than rate matched German sentences (their L1), as evidenced not only by higher speed judgments (Experiment 3) but crucially also in a higher proportion of subsequent long vowel responses (Experiments 1 and 2). This biasing effect of the language of the carrier sentence shows that the FL effect impacts online speech comprehension in an *implicit rate perception* task.

Moreover, this FL effect was consistently modulated by participants’ ability to understand/translate the FL sentences: German participants with lower Dutch translation scores showed even more of a bias towards the long vowel /a:/ after Dutch sentences than participants with higher translation scores. This modulating effect of participants’ ability to understand the FL is in line with previous studies testing *explicit rate perception* (Schwab and Grosjean, 2004; Schwab, 2014). It corroborates the interpretation that the /a:/ bias after Dutch sentences in German listeners is really related to the language in which the carrier sentences were produced and not to other acoustic aspects of the Dutch sentences.

However, the Dutch listeners tested in the present study did not show empirical support for the FL effect. Experiments 1 and 2 both revealed that Dutch listeners reported *fewer* /a:/ responses after German than after rate matched Dutch carrier sentences, contrary to our predictions. However, the interpretation that this suggests that German sounds *slow* to Dutch ears is not supported by the outcomes of Experiment 3, showing no difference in *explicit rate judgments* of Dutch and German.

At this point, we lack an accurate explanation for why the Dutch listeners reported *fewer* long vowel responses after German carrier sentences. Potential differences between Dutch and German in phonotactic probabilities of /a/ and /a:/, or typical vowel length, are unlikely to explain the unexpected variation between groups because our participants had (very) little experience with the FL, and, as such, cannot be assumed to have been familiar with such fine-grained phonological language variation. However, the present results highlight the value of using symmetrical experimental designs; that is, testing two different participant groups listening to both languages (cf. Pfitzinger and Tamashima, 2006). Without such fully crossed designs, we would either have found contradictory evidence (in the Dutch case) or would have overgeneralized the experimental findings (in the German case), especially since results were replicated across three experiments with three different samples of participants per language. Moreover, we would like to point out that, in Experiment 2, there was a modulating effect of FL proficiency on the unexpected Language effect in the Dutch group. That is, Dutch listeners with lower German translation scores showed more of a bias towards /a:/ after German sentences. This observation points to the role of FL proficiency in our Dutch and German participant samples.

Particularly, our Dutch participants consistently showed higher translation and self-rated proficiency scores in German than our German participants did in Dutch. This is not too surprising considering the fact that the Dutch participants were recruited in Nijmegen, close to the German border and with a considerable proportion of German students at the university. Dutch participants were hence not only familiar with German but also German accented Dutch. Most of the German participants, in contrast, were recruited in Munich – far from the Dutch border – and with little contact to the Dutch language or Dutch accented German. Although the relatively high FL proficiency in the Dutch groups cannot explain why Dutch listeners reported *fewer* long vowel responses after German speech, the asymmetry in proficiency across the two population samples may account for why support for the FL effect was found in the German samples, but not in the Dutch samples. Similar asymmetries between listener groups are likely hard to avoid for other language pairs. Choosing two closely-related languages allowed us to control for most factors pertaining to language structure. The effects of native language as well as the modulation of the effect by proficiency, however, lend support for the role of ease of processing in the effect. Future studies may specifically target participant samples at a range of different proficiency levels, or even experimentally test the modulating effect of FL exposure, for instance, through training studies.

As for the wider cognitive implications of the effect, the role of the ability to understand the FL matches with other findings on the underlying mechanisms of processing speaking rate more generally. Bosker et al. (2017) demonstrated that a carrier sentence is perceived as faster if listeners are taxed by high relative to low cognitive load required for a concurrent visual search task. This supports suggestions that FLs sound fast because they are harder to process; that is, words are harder to segment out of the continuous speech stream (Snijders et al., 2007). Similarly, Bosker and Reinisch (2015) showed that sentences spoken with a foreign accent that are supposedly harder to process than native speech are perceived as faster than native speech. Both studies used implicit rate normalization tasks as in the present study.

In summary, this study has demonstrated that the common impression that foreign speakers talk fast impacts online speech comprehension, particularly in the form of variation in phonetic categorization. This observation carries implications for language learners. We show that the FL rate effect not only impacts overall subjective impressions of foreign speech, but may actually influence language learners’ perception of segments in the FL.

## Author Contributions

HB and ER planned the experiments, collected and analyzed the data, interpreted the results, and wrote the paper.

## Conflict of Interest Statement

The authors declare that the research was conducted in the absence of any commercial or financial relationships that could be construed as a potential conflict of interest.

## Footnotes

1 In the language learning literature, the term ‘proficiency’ is typically only used for second language learners; not for listeners who are entirely unfamiliar with a particular FL. In contrast, we use the term ‘proficiency’ to refer to the ability to understand words in the FL, even if the FL has not been learnt in any way by most of our participants.

2 When we replace the predictor Translation Accuracy in the models of Experiments 1 and 2 with self-reported FL ratings (as used here in Experiment 3), the same interactions are observed as reported previously.
