# Supplementary material for: Foreign Languages Sound Fast: Evidence from Implicit Rate Normalization
Source: Front Psychol. 2017 Jun 28;8:1063. doi: 10.3389/fpsyg.2017.01063 (PMC5487441; doi:10.3389/fpsyg.2017.01063)
Supplement: Supplementary file 1 [file Data_Sheet_1.docx]

# Appendix

Table S1. German and (paraphrased) Dutch carrier sentences (matching syllable count) with English translations underneath.

|  | German | Dutch | syllable count |
| --- | --- | --- | --- |
| 1 | *Im Kreuzworträtsel suchten sie den Begriff*  "In the crossword puzzle, they sought the term" | *In de kruiswoordpuzzel zochten ze de term*  "In the crossword puzzle, they sought the term" | 11 |
| 2 | *Jörg tut sich schwer mit dem Wort*  "Jörg has trouble with the word" | *Piet heeft moeite met het woord*  "Jörg has trouble with the word" | 7 |
| 3 | *Der Text endete mit dem Wort*  "The text ended with the word" | *De tekst eindigde met het woord*  "The text ended with the word" | 8 |
| 4 | *Sie vermied in ihrem Text den Begriff*  "In her text she avoided the term" | *Zij vermeed in de tekst steeds het begrip*  "In the text she avoided the term" | 10 |
| 5 | *Der Stotterer mühte sich mit dem Wort*  "The stutterer struggled with the word" | *De spreker struikelde over het woord*  "The speaker struggled with the word" | 10 |
| 6 | *Im Wörterbuch steht die Bedeutung von*  "In the dictionary is the meaning of" | *Het woordenboek geeft uitleg bij het woord*  "The dictionary explains the word" | 10 |
| 7 | *Es gibt mehrere Synonyme für*  "There are several synonyms for" | *Er zijn meerdere synoniemen voor*  "There are several synonyms for" | 10 |
| 8 | *Bis gestern wählte Susi immer den Begriff*  "Until yesterday, Susi always chose the term" | *Tot gisteren koos Susie telkens voor de term*  "Until yesterday, Susie always chose the term" | 12 |
| 9 | *Wir hörten neue Theorien zur Entstehung des Wortes*  "We heard new theories about the development of the word" | *Wij hebben ook nieuwe theorieën gehoord over het woord*  "We have also heard new theories about the word" | 16 |
| 10 | *Die Kinder stritten über die Bedeutung des Wortes*  "The children quarreled over the meaning of the word" | *De kinderen voeren een discussie over het woord*  "The children have a discussion over the word" | 14 |
| 11 | *Er suchte die Übersetzung des Wortes*  "He looked for the translation of the word" | *Hij zoekt een geschikt synoniem voor het woord*  "He looks for a suitable synonym of the word" | 11 |
| 12 | *Es gibt sicher noch vier bessere Wörter für*  "There are definitely four better words for" | *Er zijn zeker nog vier betere woorden voor*  "For sure, there are four better words for" | 12 |
| 13 | *Sie gibt ihrem neuen Buch den Titel*  "She gives her new book the title" | *Zij geeft zelf het nieuwe boek de titel*  "She herself gives the new book the title" | 10 |
| 14 | *Im Märchenbuch nennt sich der Held*  "In the fairytale book the hero calls himself" | *In het sprookjesboek heet de held*  "In the fairytale book the hero is called" | 8 |
| 15 | *Die besten Lieder des Komponisten enden mit dem Wort*  "The best songs of the composer end with the word" | *Het beste lied geschreven door de componist eindigt met*  "The best song written by the composer ends with" | 15 |
| 16 | *Sie beschrieben die Bilder mit dem Wort*  "She described the images with the word" | *Ze beschreven de beelden met het woord*  "They described the images with the word" | 10 |
| 17 | *Nächste Woche gibt der Komiker die Vorstellung mit dem Titel*  "Next week, the comedian will give his performance with the title" | *Volgende week geeft de komiek twee voorstellingen met de titel*  "Next week, the comedian gives two performances with the title" | 17 |
| 18 | *Der dritte Begriff im Wörterbuch ist*  "The third term in the dictionary is" | *De derde term in het woordenboek is*  "The third term in the dictionary is" | 10 |
| 19 | *Sie gewinnt die Rätselrunde mit dem Begriff*  "She wins the quiz round with the term" | *Ze heeft de quizronde gewonnen met de term*  "She has won the quiz round with the term" | 12 |
| 20 | *Im Folgenden benutzt Georg nur noch den Begriff*  "In the following, Georg only uses the term" | *In het vervolg gebruikt Sjors enkel nog het begrip*  "In the following, Sjors uses only the term" | 13 |
| 21 | *Sie liest die Geschichte mit dem Titel*  "She reads the story with the title" | *Zij lezen het sprookje met de titel*  "She reads the story with the title" | 10 |
| 22 | *Ihr bester Freund hieß …*  "Her best friend is called ..." | *Zijn beste vriend heet*  "Her best friend is called" | 5 |
| 23 | *Die Kinder riefen den Hund immer …*  "The children always called the dog" | *De kinderen noemden de hond steeds*  "The children always called the dog" | 9 |
| 24 | *Die Säule ist mit komischen Äußerungen beschmiert wie …*  "The pillar is smeared with strange utterances such as" | *De zuilen zijn stuk voor stuk besmeurd met de komische tekst*  "The pillars are all smeared with strange utterances such as" | 15 |
| 25 | *Yvonne beschrieb die Person mit dem Begriff*  "Yvonne described the person with the term" | *Yvonne beschreef de personen met de term*  "Yvonne described the person with the term" | 12 |
| 26 | *Die Königin beginnt ihre Lesung mit dem Wort*  "The queen started her reading with the word" | *De koning is zijn lezing begonnen met de term*  "The king has started his reading with the term" | 13 |
| 27 | *Ich wusste es nicht und googelte den Begriff*  "I didn't know and googled the term" | *Ik wist het niet dus google-de ik toen het woord*  "I didn't know so I then googled the word" | 12 |
| 28 | *Sie benennen diese Dinge mit*  "They label the things with" | *Ze noemen zoiets ook wel eens een*  "They sometimes call these things" | 9 |
| 29 | *Wenn ich dir helfen soll ruf*  "If I can help you, call" | *Voor mijn hulp roep je gerust*  "For my help, feel free to call" | 7 |
| 30 | *Die gewinnende Skulptur hieß*  "The wining sculpture was called" | *En het winnende kunstwerk heet*  "The winning artwork is called" | 8 |
